# OpenSAFELY: a platform for analysing electronic health records designed for reproducible research

**DOI:** 10.1002/pds.5815

**Published:** 2024-06-01

**Authors:** Linda Nab, Andrea Schaffer, William Hulme, Nicholas J DeVito, Iain Dillingham, Milan Wiedemann, Colm D Andrews, Helen Curtis, Louis Fisher, Amelia Green, Jon Massey, Caroline E Walters, Rose Higgins, Christine Cunningham, Jessica Morley, Amir Mehrkar, Liam Hart, Simon Davy, David Evans, George Hickman, Peter Inglesby, Caroline E Morton, Rebecca M Smith, Tom Ward, Thomas O’Dwyer, Steven Maude, Lucy Bridges, Ben FC Butler-Cole, Catherine L Stables, Pete Stokes, Chris Bates, Jonny Cockburn, Frank Hester, John Parry, Krishnan Bhaskaran, Anna Schultze, Christopher T Rentsch, Rohini Mathur, Laurie A Tomlinson, Elizabeth J Williamson, Liam Smeeth, Alex Walker, Sebastian Bacon, Brian MacKenna, Ben Goldacre

**Affiliations:** 1 Bennett Institute for Applied Data Science, Nuffield Department of Primary Care Health Sciences, University of Oxford, OX2 6GG, UK; 2 TPP, TPP House, 129 Low Lane, Horsforth, Leeds, LS18 5PX, UK; 3 London School of Hygiene and Tropical Medicine, Keppel Street, London WC1E 7HT, UK

## Abstract

Electronic health records (EHRs) and other administrative health data are increasingly used in research to generate evidence on the effectiveness, safety, and utilisation of medical products and services, and to inform public health guidance and policy. Reproducibility is a fundamental step for research credibility and promotes trust in evidence generated from EHRs. At present, ensuring research using EHRs is reproducible can be challenging for researchers. Research software platforms can provide technical solutions to enhance the reproducibility of research conducted using EHRs. In response to the COVID-19 pandemic, we developed the secure, transparent, analytic open-source software platform OpenSAFELY designed with reproducible research in mind. OpenSAFELY mitigates common barriers to reproducible research by: standardising key workflows around data preparation; removing barriers to code-sharing in secure analysis environments; enforcing public sharing of programming code and codelists; ensuring the same computational environment is used everywhere; integrating new and existing tools that encourage and enable the use of reproducible working practices; and providing an audit trail for all code that is run against the real data to increase transparency. This paper describes OpenSAFELY’s reproducibility-by-design approach in detail.

## Background

Electronic health records (EHRs) and other administrative health data are increasingly used in research to generate evidence on the effectiveness, safety, and utilisation of medical products and services and inform public health guidance and policy. Broadly speaking, reproducibility refers to the ability of independent investigators to obtain the same findings when applying the same design and operational choices to the same data source. Reproducibility is a fundamental step for research credibility and facilitates reuse of shared code, improving overall timeliness, quality, trust and ultimately impact of research generated from EHRs.^1–3^ Despite the importance of reproducible, transparent research, several studies have shown published research using EHRs can be challenging to reproduce.^4,5^

The REPEAT initiative set out to reproduce results for 150 epidemiologic studies published in peer-reviewed journals using the same healthcare databases as the original investigators. The majority of research could be closely reproduced, but a subset could not: of the 150 studies reproduced, 10 extreme outliers were identified based on the difference between the original and reproduction effect sizes.^4^ Barriers identified by the investigators included ambiguous and non-transparent reporting of data processing, design and analytic choices in the original studies. In another attempt to reproduce 11 papers featuring longitudinal data analyses, it was found that reproduction was difficult in most cases and required reverse engineering of results or contacting the authors.^5^ The main barrier reported here was the unavailability of source code.

Research software platforms can offer technical solutions that mitigate these barriers by standardising data preparation and computational environments, enforcing public sharing of research materials, and enabling and encouraging researchers to incorporate reproducible working practices in their workflows. In response to the COVID-19 pandemic, we developed the secure, transparent, open-source software platform OpenSAFELY.^6^ OpenSAFELY was designed with reproducible research in mind. OpenSAFELY now operates across primary care records of >99% of the population of England with the full support of NHS England as a national secure data environment.

In this paper, we discuss how reproducibility in the context of research using EHRs differs from other research disciplines and introduce the research software platform OpenSAFELY. We describe common technical challenges in reproducible research using EHRs, and show how a platform like OpenSAFELY mitigates barriers to reproducible research by integrating existing and new technical tools in research workflows and encouraging cultural change.

## Reproducibility in the context of research using EHRs

Reproducibility and other terms such as replicability and repeatability are ambiguously defined in literature and inconsistently used in different scientific disciplines.^7^ Generally, the terms are used in relation to the concept of one experiment or study confirming the results of another.^8^ Despite efforts to unify the use of these terms, lack of consensus persists across disciplines.^9^

A recurring element in definitions for reproducibility (Box 1) is ‘same data’. The primary purpose of EHRs is to facilitate safe and efficient healthcare delivery. The use of EHRs for research is secondary. Preparing and transforming EHRs to a dataset ready to be analysed involves many decisions, often implemented in thousands of lines of code. This is very different from what is typically needed for preparing a dataset primarily collected for research for analysis, and this brings unique reproducibility challenges.^1^

**Figure 1:**
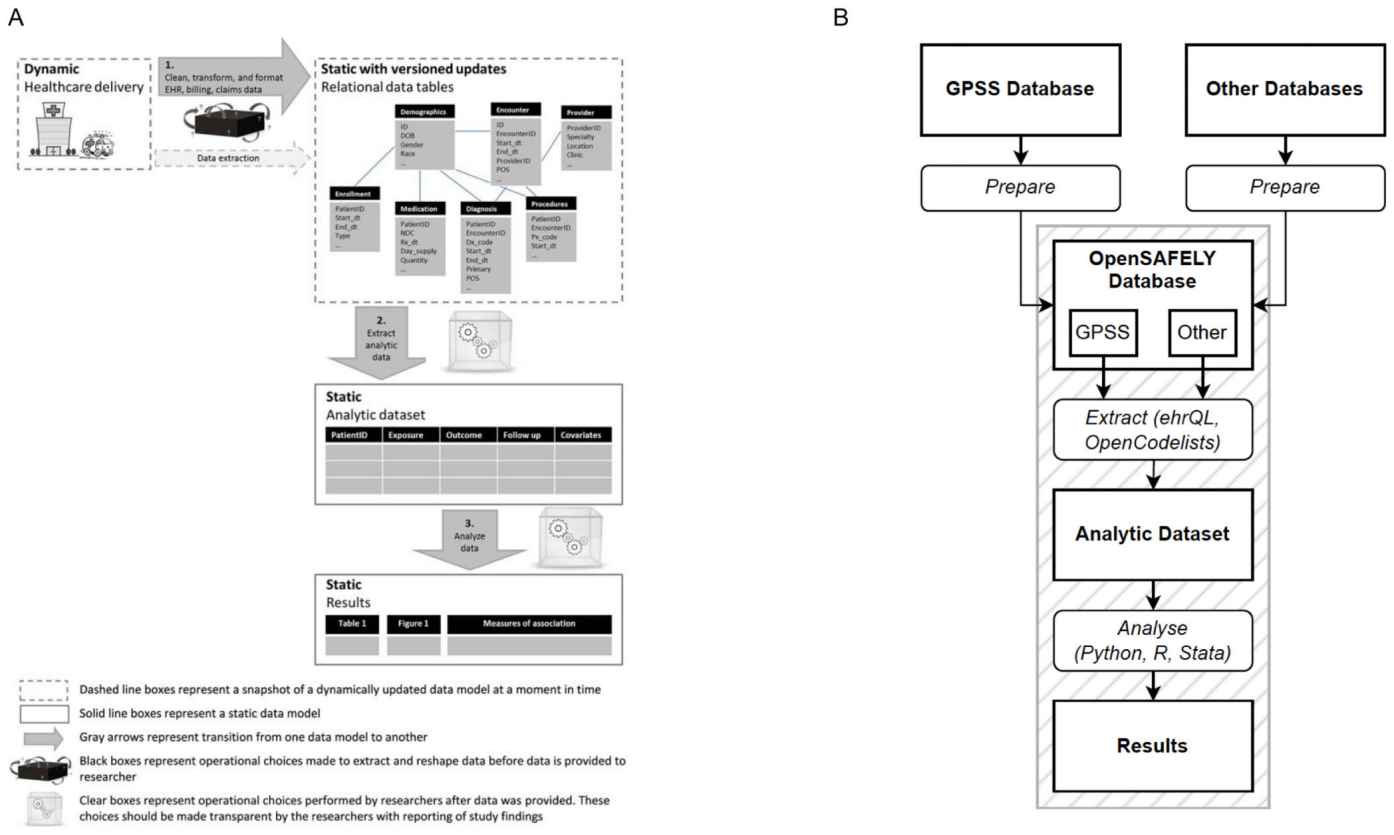
Overview of transformations from health care delivery to analysis results in research using linked electronic health records. Transformations in a general research setting (panel A, figure adapted from “Reporting to Improve Reproducibility and Facilitate Validity Assessment for Healthcare Database Studies V1.0” by Wang et al.^1^, licensed under CC BY 4.0), and transformations in OpenSAFELY* (panel B). Abbreviations: GPSS: general practice system supplier; ehrQL: Electronic Health Records Query Language. * This diagram represents a simplified overview of conceptual levels of data identifiability and data preparation in the OpenSAFELY platform from the perspective of a platform user. A detailed description of the tiered structure of data in OpenSAFELY, including data controllership and accessibility, is found in the Supplemental Material.

Wang et al. described three transformations of data from healthcare delivery to analytic results in the context of research using EHRs, depicted in Panel A of Figure 1.^1^ In terms of the transformations described by Wang et al. we focus on the reproducibility of steps 2 and 3, that is, the transformation from a *healthcare research database* to a *analysis-ready dataset* and the transformation from that dataset to *analytic results*, respectively. We acknowledge that step 1, the understanding of how a source *healthcare delivery database* is cut, cleaned and pre-processed to create a healthcare research database, is also an important step in reproducible research using EHRs. This data pre-processing step is however often done by the EHR vendor and therefore beyond the control of researchers, as is, for example, the case in OpenSAFELY. We believe that reproducibility of this step could be promoted by only doing strictly necessary steps (e.g., disclosive steps) in private and pushing as many transformations as possible to step 2.

More specifically, we focus on the *computational* reproducibility of the two-step transformation from a healthcare research database to analytic results. Computational reproducibility focuses on whether research is *capable of being checked* because the data, code and methods of analysis are available to independent researchers.^8^ This is in contrast to *independent* reproducibility, which focuses on effective communication of critical design and analytic choices, needed to ascertain intended scientific conclusions and validate analytic choices.^4^ While effective communication of choices made in the transformation from the healthcare research database to analytic results to achieve independent reproducibility is important, our focus is on how platforms like OpenSAFELY improve the computational reproducibility of EHR research by offering technical solutions and encouraging cultural change.

### OpenSAFELY: a platform designed with reproducible research in mind

OpenSAFELY is currently deployed in the data environments of electronic health record system suppliers TPP and EMIS, granting safe access to the full primary care EHRs of >99% of the population in England. Data are linked in situ with other health and administrative datasets, including hospital activity, death records, and various disease and treatment registries. Deploying OpenSAFELY in an EHR data centre creates a trusted research environment (TRE) or secure data environment through which researchers interact with the data. The terms TRE and secure data environment can be used interchangeably in this context. In this paper, we will use the word TRE.

A TRE allows users to work with sensitive data remotely, rather than downloading copies onto a local machine. Researchers can extract and download the answers from their analyses – such as results tables, or graphs – but individual patients’ data and other disclosive information always stays within the TRE.^13^ As such, data stored and accessed on TREs cannot be shared. Despite this restriction, in a typical TRE the analysis-ready datasets are directly accessible and the healthcare research database can still be interacted with by researchers from authorised organisations, usually through a “*remote virtual desktop*”. Like other TREs, OpenSAFELY is designed around the Five Safes framework for providing safe and efficient data access (Box 2).^14^ However, an OpenSAFELY TRE departs from this typical TRE design: researchers do not need to view the healthcare research database or analysis-ready dataset to conduct their analyses. Instead, they use the OpenSAFELY tools to transform the healthcare research database into an analysis-ready dataset, and to execute data analysis and visualisation code against that dataset; they view only the analytic results of those analyses.

The OpenSAFELY platform is highly secure. The underlying patient data, and intermediary analysis datasets are arranged in a tiered structure (or “levels”) specifically designed to reduce the disclosiveness of the information. A detailed description of the tiered structure, including data controllership and accessibility, is found in the Supplemental Material. For simplicity, we will here distinguish between three conceptual levels of data identifiability and data preparation from the perspective of a platform user: the ‘OpenSAFELY database’, ‘analytic dataset(s)’, and ‘results’ (Figure 1, Panel B).

The OpenSAFELY database is prepared by the EHR vendor (on behalf of GP practices) and contains a subset of the complete patient record from primary care, not including free text; this data is pseudonymised and de-identified at source and then linked in situ to similar prepared pseudonymised and de-identified external datasets (e.g., from hospital systems). The OpenSAFELY database is segmented and data from GP practices and external datasets are only brought together at the level of the ‘analytic dataset’. In the deployment of OpenSAFELY at TPP, the pseudonymised primary care data is rebuilt every week, reflecting changes to the raw patient data at the EHR vendor’s data centre. External datasets have varying update schedules, ranging from weekly to ad-hoc requests for updates.^15^

The ‘analytic dataset(s)’ is the analysis-ready dataset extracted from the OpenSAFELY database and created by the platform user using OpenSAFELY tools. The ‘results’ refer to the aggregated output resulting from the platform user’s analysis scripts run on the analytic dataset, such as tables and graphs. Researchers can view these results in the TRE on a “remote virtual desktop”, but do not have access to the OpenSAFELY database or analytic datasets. When a researcher is ready to disseminate results, they propose the results for release outside the TRE; these are manually checked and only safe outputs (Box 2) are released.

## Challenges of reproducible research using EHRs

One of the challenges of ensuring that research using EHRs is computationally reproducible is the inability to share the sensitive medical information of individual patients which is used. To protect privacy, laws are put in place such as the EU’s General Data Protection Regulation and the US Health Insurance Portability and Accountability Act, prohibiting sharing data obtained from EHRs and therefore hindering checks on reproducibility.^16^ A recent meta-analysis of the medical and health sciences showed a prevalence of declared public data availability of 8% which did not correspond to actual data sharing, being even lower with a prevalence of 2%. The low prevalence of data sharing in the medical and health sciences are likely to be largely associated with privacy constraints, explaining why data sharing was more prevalent in other disciplines like ecology and computer sciences.^17^

Not only do EHRs contain sensitive information, EHR databases are often dynamic, and historical data are sometimes retrospectively updated, or expanded upon, by the data provider. As an example, the OpenSAFELY-TPP database is rebuilt weekly. This allows timely research of health data but at the same time impedes exact reproduction of the analytic dataset after the database is rebuilt: patients can leave or enter the database by changing their practice, and historical data can be corrected or updated by practitioners. The REPEAT initiative studied the sensitivity of results to updates of EHR databases. While most of the studies in the REPEAT initiative could be closely reproduced using updated data, they observed that in one of the studies reproduced, a shift in the EHR source data in different data versions played a large role in finding a larger, older and sicker analytic reproduction cohort, resulting in difficulties reproducing outcome risks and rates of that study.^4^

Besides data sharing and consistency, the transformation from a healthcare research database to an analysis-ready dataset (step 2, Figure 1 panel A) and from an analysis-ready dataset to analytic results (step 3, Figure 1 panel A) are complex steps involving many decisions: making these transformations reproducible is challenging.

### Transforming a research database into a dataset

The transformation from a healthcare research database into an analysis-ready dataset is often done in siloes, and code documenting this step is rarely (openly) available.^3^ Failure to publicly share a project’s programming code obstructs computational reproducibility across all facets of the project. On some occasions, data might even be changed in the database directly by researchers. These manual data manipulation steps are hard to reproduce, especially if they are not documented.^18^

Reshaping a healthcare research database into an analysis-ready dataset also often involves codelists: a list of codes for clinical events or demographic characteristics used to map EHR data to variables used in an analysis. For example, in a study looking at patients with hypertension you would need a codelist to define the various types and subtypes of hypertension, while excluding terms that are not relevant to your specific research question. Publishing a study’s codelists is necessary for accurate reproduction: the REPEAT initiative found a 26% difference in sample size between original study cohort and the reproduction cohort partly because the codes used to define chronic obstructive pulmonary disease were not specified and needed to be recreated by the study team.^4,19^ However, publishing a study’s codelists is not sufficient to ensure reproducibility due to a phenomenon that we have coined as *codelist rot*.^20^ Codelist rot refers to a class of problems that causes codelists to become outdated or invalid. Coding systems are frequently updated with addition of new concepts, retirement of old concepts, and changes to existing concepts (including changing the unique code for a given concept). As an example, NHS Dictionary of medicines and devices (dm+d) is updated weekly.^21^ Codelists need to be built using the same version of the coding dictionary as the data. The more frequently the data are updated, the more of an impact codelist rot has on ongoing research. Simultaneously, it also poses a problem for reproducing any EHR study if an updated version of the database is used to attempt reproduction of an old study. Not documenting strategies used to build codelists and the version of the coding dictionary used when the codelist was built hinders reproducibility.

### Transforming a dataset into analytic results

Once the healthcare research database is transformed into a dataset ready to be analysed, the next step in the EHR research pipeline is the analysis undertaken to produce the results of the study. An essential ingredient of a computationally reproducible analysis is the availability of the analysis code used to obtain the results.^22^ A recent meta-analysis found that public code sharing was persistently low in the medical and health sciences with a prevalence of available analytic code of less than 0.5%.^17^

Sharing code comes with challenges. As with data, patient privacy in publicly available code must be considered at all times. This can be at risk if, for example, there is any appearance of research results in analysis scripts or the comments and documentation therein. When analytic scripts are written and developed inside a TRE, sharing code can be laborious as all scripts have to go through a manual process to check if the code is non-disclosive and safe before being exported from the TRE. Sharing all code underlying a research project is often regarded as unrealistic in these settings.^13^

### Sharing code alone is not enough

In both transformations outlined above, lack of publicly shared code obstructs computational reproducibility. Sharing code is a minimal standard, but does not ensure reproducibility, even if data are also shared: open is not enough.^23^ A large-scale study on research code quality and execution analysed more than 2000 research studies who deposited their study materials on the Harvard Dataverse repository and found that 74% of files written in the R software language failed to complete without error on re-execution. Even after removing absolute file paths, standardising file encoding and identifying and importing used libraries to set up a proper execution environment, 56% failed to complete without errors. This shows that many errors can be prevented with good coding practices, but also shows that successful re-executions require much more than just sharing programming code and data. Another important ingredient for a successful code re-execution is the availability of the third party software or libraries the code depends on (e.g. R, Python, Stata). Reproducibility is hindered if this software is not specified or made available. As such, use of free and open-source software is recommended whenever possible.^24^ Of note, re-executable code does not ensure computational reproducibility, but can be seen as a minimal standard: output of a re-executed analysis can still quantitatively differ from the results reported.

### Non-technical barriers

Funders and publishers of medical research have been increasing the pressure on medical researchers to adopt open science practices.^25–27^ Producing reproducible research requires technical skills, time and resources and causes concerns around intellectual property, yet is undervalued and largely uncredited.

## The OpenSAFELY toolkit for reproducible research

Platform users (typically researchers) interact with the OpenSAFELY platform through a number of tools that aim to create a coherent, transparent, and reproducible workflow, some of which were specifically designed for OpenSAFELY: the electronic health records query language (ehrQL), OpenCodelists, OpenSAFELY Jobs and the OpenSAFELY Docker images (Box 2).

ehrQL is a query language custom built to retrieve records from the OpenSAFELY database. It was designed by the OpenSAFELY team specifically for use with EHR data but is portable to other settings. Besides portability, ehrQL was designed with a focus on readability (people without deep technical knowledge of ehrQL should be able to read and understand ehrQL) and composability and reusability (ehrQL queries can be split into small components which can then be re-used between studies, reducing error-prone and duplicative work).^28^ Platform users define the study population and select variables of interest with the query language (referred to as the “dataset definition”) to generate the analytic datasets. ehrQL allows users to run a query against multiple databases, without needing to spend time investigating the implementation details of each database. This means that, for example, in the deployment of OpenSAFELY in TPP and EMIS, the same queries can be run against both healthcare research databases even though the underlying database schemas are different. Not only does this result in a dataset definition that is easier for another user to understand, it also means that the same concept is expressed in the same way when run against multiple databases. All ehrQL queries run against the OpenSAFELY database are committed to GitHub and publicly logged on the OpenSAFELY Jobs website (https://jobs.opensafely.org/) (Box 2).

Platform users create their dataset definition using ehrQL which often relies on codelists: a collection of all relevant clinical codes which define a concept of interest. The OpenSAFELY platform integrates with a tool for building, sharing and version controlling codelists, along with the criteria used to build them, in public: OpenCodelists (https://www.opencodelists.org/) (Box 2). OpenCodelists is integrated in the OpenSAFELY workflow, making it easy for users to adapt reproducible working practices around the use of codelists. Codelists can be referred to using the URLs on OpenCodelists, which makes it easy to update codelists to reflect updates of coding systems. OpenCodelists uses a version control system that helps track the development and changes to codelists over time while also helping to keep codelists up-to-date in the face of updates of a coding system. OpenCodelists saves the search strategies used for the construction of a codelist. This helps in understanding the logic used for the construction of the codelist and reconstruction of the codelist in future. OpenCodelists replaces manually managing codelists in tools like Microsoft Excel which can cause problems by automatically transforming codes in unwanted fashion. For example, SNOMED CT codes are large integers that Microsoft Excel automatically transforms into floating-point numbers, making these codes unusable by, for instance, rounding trailing digits to 0s.^29^

Platform users do not have direct access to the OpenSAFELY database or analytic dataset. This way of working is only practical if users have some way of understanding the data structure to develop their code, and confirm that it works as expected. To support this, when queries are designed in ehrQL, dummy data (Box 2) are generated that has the same structure as the real analytic dataset, but none of the disclosive risks. This allows development and testing of study code without access to the real analytic dataset.

Platform users develop their code on dummy data outside of the TRE, ensuring all code is always non-disclosive and development of the analysis is conducted independent of the real data. By developing and writing code outside the TRE, sharing code does not rely on laborious manual checks before being exported from the TRE. The removal of privacy risks from data management and analysis code frees up OpenSAFELY code for sharing and reuse under open licences. This means that there is no information governance or privacy barrier to users sharing code for others to review, critically evaluate, improve, and re-use, wherever they wish. On top of that, anyone can use the ehrQL code to generate their own dummy dataset on which they can re-execute the full analysis. Publishing simulated datasets along with code was proposed as a pragmatic approach for reproducible research with sensitive data ^30^: OpenSAFELY makes this easy because the full analysis is re-executable using dummy data by design, depositing one specific dummy dataset and associated results is straightforward.

Using the automatically generated dummy data in each project, anyone can rerun an OpenSAFELY analysis using basic software: an online environment where the needed software is already installed for you (GitHub Codespaces, Box 2) or by installing the required software to your computer. The OpenSAFELY platform ensures the same computational environment is used everywhere by providing Docker images (Box 2) and making sure the same library versions of packages are used everywhere. This makes sure code is always re-executable as anyone can reproduce the computational environment the code was developed and executed in.

GitHub (Box 2) is integrated in OpenSAFELY: any code run against the real data has to be committed and pushed to GitHub. Sharing code is therefore not a choice, but a requirement. It is accepted that some code may remain private while an analysis is in development. However, all code is published when the results of the analysis are shared (e.g., when a paper is preprinted or published). Even if a project remains ongoing, users agree to open up their code no later than 12 months after any code has been executed against the real data. As research code is rarely shared, this creates a transparency requirement for OpenSAFELY projects that goes beyond the current norms of the field. This allows audit, inspection, and reuse of all code created for use on the platform.

Not only is GitHub a tool for sharing code, it also allows for iterative development with version control, and code review. The integration of GitHub in OpenSAFELY encourages and enables users to integrate these reproducible working practices into their workflows. The integration also allows users to test the full analytic pipeline through GitHub Actions everytime they push new code to GitHub (Box 2). This makes sure errors are caught early, without the need of running it on the real data, and enables users to check and guarantee their code is always in a re-executable state.

OpenSAFELY Jobs (https://jobs.opensafely.org/) is the main web interface for most of the interactions a user has with the platform (Box 2). Users can only run code against the real data at arm’s length, via OpenSAFELY Jobs. This means that there is a public log of every line of code run against the real data, ever. This encourages clear, upfront, hypothesis-driven design and discourages data dredging: testing multiple hypotheses using a single dataset by exhaustively searching. In OpenSAFELY, users package code to run on the real data in an action: a blueprint for what OpenSAFELY needs to do. A research study is typically composed of a set of actions, which are grouped into a project pipeline. In each action, the code that needs to be run is specified, thereby listing any dependent actions and defining the expected outcomes. Most studies start with an action with the “dataset definition”, which extracts the analytic dataset from the OpenSAFELY database. Downstream actions may reshape or model the analytic dataset. An OpenSAFELY project pipeline is specified in a YAML file, which is a structured, human-readable text file. This file captures the correct execution sequence of the code and specifies dependencies of one action on another action. Because there is no other way to run code against real data than to define it as an action in the project pipeline, this creates the requirement for OpenSAFELY projects to define the sequence of actions and corresponding dependencies by design. Besides the execution sequence, the YAML file specifies which Docker image (Box 2) and version was used to run the script. Old Docker images are kept available, meaning that a script relying on an older version of, for example, R will remain re-executable.

## Discussion

### Summary

Reproducibility is a fundamental step for research credibility and promotes trust in evidence generated from EHRs. At present, ensuring research using EHRs is reproducible can be challenging for researchers. Ensuring reproducibility of research is challenging and requires time, resources and skills, most of which researchers are typically not trained for. Strong research software platforms can help make adopting reproducible working practices in research workflows easy and takes the weight off of the shoulders from researchers. We developed the data analytics platform OpenSAFELY in response to the COVID-19 pandemic on behalf of NHS England. The platform was designed with reproducible research in mind and allows secure analysis of pseudonymised primary care EHRs from England in near real-time within the EHR vendor’s highly secure data centre. OpenSAFELY aims to make reproducible research the norm by: standardising key workflows around data preparation; removing barriers to code-sharing in secure analysis environments; enforcing public sharing of programming code and codelists; ensuring the same computational environment is used everywhere; integrating new and existing tools that encourage and enable the use of reproducible working practices; and providing an audit trail for all code that is run against the real data to increase transparency.

### Strengths and weaknesses

OpenSAFELY software ensures that all research activity is publicly logged and all code for data management and analysis is shared, under open licences and by design, for scientific review and efficient reuse. Through OpenSAFELY, reproducible working practices are integrated in day-to-day work and are not an afterthought. Some of these working practices are enforced by the system (e.g., standardising data preparation and the computational environment, open code sharing, public log of all code run on the real data in OpenSAFELY Jobs, formalising dependencies between analysis scripts via project pipelines) some are greatly enabled (e.g., checks for code being in re-executable state through GitHub Actions), and some are strongly encouraged (e.g., publication of codelists on OpenCodelists, code review).

OpenSAFELY maintains a flexible approach by not enforcing adoption of all reproducible working practices to its users (e.g., code reviews).. While there is a risk non-mandatory practices might not get adopted by all users, we believe this flexibility is needed and OpenSAFELY’s open-by-design approach makes it transparent what practices are adopted and what might be improved by, for example, offering additional training or developing new tooling. We believe mandatory open code sharing is a step in the right direction, but not a panacea. Open code does not guarantee quality, as evidenced in a large-scale study on research code quality, finding 56% of research projects not being re-executable even though code was shared^24^; for this reason OpenSAFELY also focuses on ensuring code is re-executable by making available the computational environment the code was developed and executed in.

An important barrier of computational reproducibility of research using EHRs is the inability to share sensitive medical information of individual patients. To protect patient’s privacy even further, a unique design feature of OpenSAFELY is that researchers only view aggregated output. Because all code is developed on dummy data and not on real data, we can ensure code is always non-disclosive and can therefore be publicly shared. However, the lack of direct interaction with the data may make problem solving or code development more challenging and time consuming for researchers. For this reason OpenSAFELY provides tooling to standardise workflows shared between studies such as data preparation and codelist development, openly shared for efficient re-use. By design, to eliminate privacy risks, the dummy data in OpenSAFELY are not synthetic data: synthetic data are usually based on real-world datasets, while dummy data are created independent of the real data.^34^ As a consequence, dummy data will not accurately represent the relationships between variables that may exist in EHRs; nor can it be used to assess up-front feasibility of research.

### Findings in Context

There have been numerous initiatives to document the prevalence of code sharing.^17^ A recent meta-analysis of meta-research studying the prevalence of code sharing showed that both declared and actual code sharing in medical and health research was <0.5% since 2016 and did not increase meaningfully over time, despite increased awareness of its importance, and increased adoption of code-sharing requirements by journals. By contrast, of all 59 completed research papers conducted using the OpenSAFELY platform across 11 different organisations in the UK indexed on PubMed on 8th Jan 2024, 100% adhered to best practice on open code sharing, by making this a standard part of the workflow through the design of the platform (Supplemental Material).

Similarly, there have been numerous other initiatives aiming to improve the prevalence of code sharing in health research. Examples include the UK Reproducibility Network ^35^, the Carpentries ^36^, and journals and funders have been starting to impose requirements around open science (e.g., BMJ, BMC, Wellcome Trust ^25–27^), which are principally based around education and audit. OpenSAFELY shows how research software platforms can achieve full adherence to open code sharing and provide solutions for common challenges of reproducible research by integrating reproducible working practices into research workflows.

Other sources for accessing UK primary care data for research include Clinical Practice Research Datalink^37^, QResearch^38^, and The Health Improvement Network^39^. However, to our knowledge OpenSAFELY is the only one to put a primary focus on reproducibility.

### Future research

OpenSAFELY has shown how a research software platform using EHRs can increase adoption of reproducible working practices in research workflows. One future area of interest is to evaluate whether there has been increased adoption of non-mandatory reproducible working practices by researchers in OpenSAFELY compared with research elsewhere - for instance, increased use of code reviews by peers, and open archiving of clinical codelists on OpenCodelists allowing version control. Similarly, it is of interest to assess whether OpenSAFELY measurably increases research efficiency across different research groups for instance due to the availability of standardised, reusable code.

### Conclusion

Reproducibility is important for research credibility and quality, and promotes trust in evidence generated from EHRs. However, adoption of practices that promote reproducibility and transparency for research involving EHRs has been slow. The reasons are multifactorial and are both technical and cultural. While culture change will be needed for everyone to fully embrace open reproducible science, we have demonstrated how OpenSAFELY provides technical facilitators that enable and encourage—and in some cases enforce—open working practices enhancing reproducibility while maintaining patient privacy. Not only does this make working openly and reproducibly easier by addressing some of the key challenges, but it normalises these practices which is a step in the right direction towards greater cultural change.

## Supplementary Material

Supplemental Material

Supplemental Material

## Box 1 Five definitions for reproducibility

The earliest use of the term ‘reproducible research’ in a scholarly publication is by Claerbout & Karrenbach, meaning “authors provide **all the necessary data and the computer code** to run the analysis again, recreating the results” (definition 1).^10^ The Turing Way adopted the terminology by Claerbout & Karrenbach and defined a reproducible result as “when the same analysis steps performed on the **same dataset** consistently produces the **same answer**” (definition 2).^11^ Closely related to the Turing Way definition, Goodman et al. defined *methods reproducibility* as: “the ability to implement, as exactly as possible, the experimental and computational procedures, with the **same data and tools**, to obtain the **same results**” (definition 3).^7^ Goodman et al. pointed out that the same results do not necessarily lead to the same answer, as not all researchers will draw the same conclusions from the same results. In their proposed lexicon, they added *inferential reproducibilit*y to cover this distinction: “the making of knowledge claims of similar strength from a study replication or reanalysis” (definition 4). Peng et al. define reproducible epidemiologic research as research of which **the analytic dataset, computer code and software environment are available**, human readable, well documented and distributed in a standardised way (definition 5).^12^

## Box 2 Glossary of OpenSAFELY tools and terms

**OpenSAFELY trusted research environment (TRE):** A TRE allows users to work with sensitive data remotely, rather than downloading copies onto a local machine. In an OpenSAFELY TRE, users are not granted access to view the healthcare research database or analysis-ready datasets: instead, users specify analyses outside the TRE using dummy data and submit these remotely using tools built into the OpenSAFELY platform and only have access to view aggregated research outputs such as tables and figures.

**ehrQL:** Both a query language custom built for querying records in the OpenSAFELY database, and a tool for extracting those datasets from that database.

**Dataset definition**: Definition of the study population and variables of interest written in, and extracted from the OpenSAFELY database by ehrQL.

**Dummy data**: Artificial dataset generated by ehrQL with the same column names and data types (e.g., boolean, categorical variable with specified categories, continuous variable) as the analytic dataset in the OpenSAFELY TRE. Dummy data helps users to develop and test their analysis code before it is run against real patient data.

**OpenCodelists**: A tool for creating and sharing codelists (https://opencodelists.org/).

**Project pipeline**: Each research study is composed of a set of actions, which are specified in a project pipeline. Most studies start with ehrQL, which extracts a dataset from the OpenSAFELY database. Downstream actions may reshape or model the data in this dataset. An OpenSAFELY project pipeline is specified in a YAML file, which is a structured, human-readable text file. This file captures the correct execution sequence of the code and specifies dependencies of one action on another action.

**OpenSAFELY Jobs**: The main web interface for most of the interactions a user has with the platform, allowing authorised users to a) run their code against real data by running actions from the project pipeline b) check the status of these code executions c) view aggregated research outputs of the analyses in the TRE and d) view safe outputs released from the TRE. It provides an audit trail for all code that is run against the real data using the OpenSAFELY platform (https://jobs.opensafely.org/).

**Five Safes:** A popular framework for designing safe and efficient data access systems widely adopted by TREs.^14^ The Five Safes include:

- Safe People: are the researchers using the data appropriately trained and aware of their role in data protection?
- Safe Projects: does the project make good use of the data? Is it lawful and in the public interest?
- Safe Data: what is the potential for individuals to be identified in the data?
- Safe Settings: are there any technical controls on access to the data?
- Safe Outputs: are there any residual risks in outputs such as tables and figures being released from the secure environment? This is most commonly addressed by requiring outputs are independently checked for risk of disclosure of patient identifiable information by two trained ‘Output checkers’.^31^

Further detail on how OpenSAFELY addresses each of these aspects of the framework can be found elsewhere.^32^

**Released outputs**: Safe outputs moved from the TRE to OpenSAFELY Jobs after being checked for disclosivity; viewable by authorised users.

**Published outputs**: Released outputs in OpenSAFELY Jobs viewable by the public after approval from the data controller (NHS England).

**Docker**: Tool for creating images and running them as containers, or reproducible computational environments. OpenSAFELY provides images for R, Python and Stata.

**GitHub**: Tool for collaborative working, supporting reproducible working practices, such as code sharing, code review and version control. GitHub is a tool used by many software engineers, freely available and has a low barrier to entry for health data researchers.^33^

**GitHub Codespaces**: Pre-configured development environments in the cloud. Rather than installing software on their own computer, a user creates and connects to a codespace that is pre-configured with the software.

**GitHub Actions**: Software automation tool. Allows actions to be run in response to other actions. For example, allows tests to be run automatically each time code is updated on GitHub.
