# Supplementary material for: OpenSAFELY: a platform for analysing electronic health records designed for reproducible research
Source: Pharmacoepidemiol Drug Saf. Author manuscript; Available in PMC 2024 Aug 15. (PMC7616137; doi:10.1002/pds.5815)
Supplement: Supplemental Material [file EMS197224-supplement-Supplemental_Material.docx]

# Supplemental Material

### Tiered data structure in OpenSAFELY

The data pipeline for OpenSAFELY operates across 4 “levels”, all residing in the secure environment of the EHR vendor. Level 1 contains pseudonymised raw event-level GP data created by GP practices inside the EHR system (and held under the data controllership of the GP practices). Level 2 contains pseudonymised raw event-level data from other sources outside of primary care (under the data controllership of NHS England). Level 3 contains the analysis-ready patient-level datasets created by the analyst using the OpenSAFELY open source tools on Level 1 and Level 2 data (under the data controllership of NHS England); Level 3 is also the environment in which the researcher’s analysis code is run on patient-level data by the OpenSAFELY tools, without the user having direct access to view the Level 3 data. Level 4 contains the aggregate output of the researcher’s analysis code, and is the environment in which users can review the logs, graphs, and tables created by their data preparation and analysis code. When the user is ready to disseminate their results, they propose tables and graphs from Level 4 for release in a publication; these are manually checked and only safe outputs (Box 2, main text) are released for publication. For more detailed information and a graphical representation of OpenSAFELY’s levels, see https://docs.opensafely.org/security-levels/.

In the deployment of OpenSAFELY at TPP, the level 1 OpenSAFELY database (of pseudonymised event-level primary care data under the data controllership of GP practices) is rebuilt every week, reflecting changes to the raw patient data at the EHR vendor’s data centre. External datasets (in Level 2, under the data controllership of NHS England) have varying update schedules, ranging from weekly to ad-hoc requests for updates.

### Audit of availability of open code for OpenSAFELY papers

We searched PubMed on January 8th, 2024 for research studies conducted using the OpenSAFELY platform. We used the search term “opensafely” without restricting to specific fields. For all papers resulting from this PubMed search, we included all original research studies requiring code and excluded papers not requiring code (e.g., commentaries). For the included papers, we looked in the published paper or supplementary material available on the journal’s website for the primary organisation of the first author and for a link to the code repository on GitHub underlying the paper’s analyses. We identified the link to the paper’s code repository by searching for “github”, “code” and “opensafely” in the paper in subsequent order. If no direct link to the code repository on GitHub was found but a more general statement was included like “all code for the OpenSAFELY platform and analysis is openly available for inspection and re-use at github.com/opensafely” or more general “all code for the full data management pipeline—from raw data to completed results for this analysis—and for the OpenSAFELY platform as a whole is available for review online”, we searched in OpenSAFELY’s organisation on GitHub for the code repository using the title of the paper or relevant keywords. For all identified code repositories on GitHub, we checked if the repository was public.

The PubMed search resulted in a selection of 74 papers, of which 15 were deemed irrelevant because they were 1) protocols; 2) commentaries or; 3) corrections to original papers. In 52 of the remaining 59 papers, a direct link was found to the code repository on GitHub. In 7 papers, a general link to OpenSAFELY’s organisation on GitHub was included or a more general statement that code of the OpenSAFELY platform is available for review online . For all 7 papers, the relevant code repository could be easily identified when searching for the paper’s title or relevant keywords in OpenSAFELY’s Github organisation. All 59 code repositories on GitHub were public. The first authors of the papers were primarily affiliated with 11 different organisations in the UK. In conclusion, of all 59 completed research papers conducted using the OpenSAFELY platform across 11 different organisations in the UK indexed on PubMed on 8th Jan 2024, 100% adhered to best practice on open code sharing.

An overview of the included and excluded papers from our search on PubMed, primary affiliations of first authors and links to the paper’s associated code repositories on GitHub can be found in the supplementary csv file.
